# MLKL deficiency attenuated hepatocyte oxidative DNA damage by activating mitophagy to suppress macrophage cGAS-STING signaling during liver ischemia and reperfusion injury

**DOI:** 10.1038/s41420-023-01357-6

**Published:** 2023-02-10

**Authors:** Jian Xu, Dongming Wu, Shun Zhou, Haoran Hu, Fei Li, Zhu Guan, Xinyu Zhan, Yiyun Gao, Ping Wang, Zhuqing Rao

**Affiliations:** 1 Hepatobiliary Center, The First Affiliated Hospital of Nanjing Medical University; Laboratory of Liver Transplantation, Chinese Academy of Medical Sciences; Research Unit of Liver Transplantation and Transplant Immunology, Chinese Academy of Medical Sciences, 210029 Nanjing, China; 2 Department of Anesthesiology, The First Affiliated Hospital of Nanjing Medical University, 210029 Nanjing, China

**Keywords:** Monocytes and macrophages, Experimental models of disease

## Abstract

Mixed-lineage kinase domain-like protein (MLKL)-mediated necroptosis has been implicated in aggravating liver ischemia and reperfusion (IR) injury. However, the precise role and mechanism of MLKL in regulating oxidative DNA damage of hepatocytes and subsequent activation of macrophage stimulator of interferon genes (STING) signaling remains unclear. In this study, we investigated the role of MLKL in regulating the interplay between hepatocyte injury and macrophage pro-inflammatory responses during liver IR injury. We found that IR increased MLKL expression in liver tissues of wild type (WT) mice. MLKL knockout (KO) attenuated liver IR injury and suppressed the activation of cGAS-STING signaling in intrahepatic macrophages, which was abrogated by STING activation with its agonist. Mechanistically, IR induced oxidative DNA damage in hepatocytes, leading to cGAS-STING activation in macrophages, which was suppressed by MLKL KO. Moreover, increased PTEN-induced kinase 1 (PINK1)-mediated mitophagy contributed to reduced oxidative DNA damage in hepatocytes and subsequent decreased activation of STING signaling in macrophages in MLKL KO mice. Our findings demonstrated a non-canonical role of MLKL in the pathogenesis of liver IR. MLKL deficiency significantly promoted PINK1-mediated mitophagy activation to inhibit oxidative DNA damage in hepatocytes, which in turn suppressed macrophage cGAS-STING activation and inflammatory liver IR injury.

## Introduction

Liver ischemia and reperfusion (IR) injury is one of the main causes of hepatic dysfunction and failure in partial hepatectomy or liver transplantation [1]. Both ischemia-induced direct injury of liver parenchymal cells and immune responses play important roles in the development of inflammatory liver injury. Damage-associated molecular patterns (DAMPs) released from stressed hepatocytes stimulate macrophages via pattern recognition receptors to trigger an immune response [2]. A better understanding of the precise regulatory mechanism of the interplay between hepatocellular injury and macrophage innate immune activation is imperative for designing therapeutic strategies to attenuate liver IR injury in patients.

Different types of cell death occur simultaneously in various liver diseases, including liver injury, metabolic liver disease, and cancer [3, 4]. Necroptosis is a regulated form of necrosis that can trigger an innate immune response by releasing intracellular pro-inflammatory components [5]. Receptor-interacting protein kinase (RIPK)1, RIPK3, and their substrate, mixed-lineage kinase domain-like protein (MLKL), are implicated in the necroptosis signaling pathway.

Cyclic GMP-AMP synthase (cGAS) has recently been identified as a DNA sensor. Upon ligand engagement, cGAS produces second messenger 2’,3’-cyclic GMP-AMP (cGAMP), which further activates stimulator of interferon genes (STING), subsequent type I interferons, as well as NF-kB signaling [6]. Moreover, cGAS can also be stimulated by self-genomic or mitochondrial DNA in inflammatory and autoimmune diseases and tumors [7]. Emerging evidence has shown that cGAS-STING signaling plays a critical role in liver diseases, including IR injury [8–10]. We previously found that mitochondrial DNA (mtDNA) from ischemia-stressed hepatocytes activates macrophage cGAS-STING signaling to promote nucleotide-binding domain and leucine-rich repeat containing protein 3 (NLRP3)-mediated inflammatory IR injury in aged livers [11, 12].

The role of MLKL in regulating liver IR injury has recently been reported [13]. Inhibition of MLKL suppresses ischemia-induced hepatocyte necroptosis in vitro [14]. MLKL-knockout (KO) decreases hepatic neutrophil infiltration and inflammation and IR injury in steatotic mice [15]. In contrast, anti-necroptosis treatment does not protect against hepatic IR injury [16]. Another study also showed that alcoholic steatosis-promoted IR injury is independent of MLKL-mediated necroptosis [17]. Moreover, MLKL has been reported to regulate macrophage phagocytosis [18] and hepatocyte autophagy [19] in a necroptosis-independent manner. Therefore, the precise role and underlying mechanism of MLKL in regulating liver IR injury needs to be further studied.

In the present study, we investigated the role of MLKL in regulating the interplay between hepatocyte injury and macrophage pro-inflammatory responses during liver IR injury. To date, the role of hepatocyte MLKL in regulating macrophage STING activation during liver IR injury has not been reported. This study will help in providing new mechanistic insights into the pathogenesis of liver IR injury. Further, this information will be beneficial for performing future research to prevent hepatic dysfunction and failure in partial hepatectomy or liver transplantations caused by liver IR injury. MLKL deficiency promoted mitophagy activation to reduce oxidative DNA injury in hepatocytes, which suppressed the activation of cGAS-STING signaling in macrophages, leading to attenuated liver IR injury. Together, we demonstrated a non-canonical role of MLKL in the pathogenesis of liver IR injury through the inhibition of mitophagy in hepatocytes.

## Results

### MLKL depletion protected the liver against IR injury

**Fig. 1:**
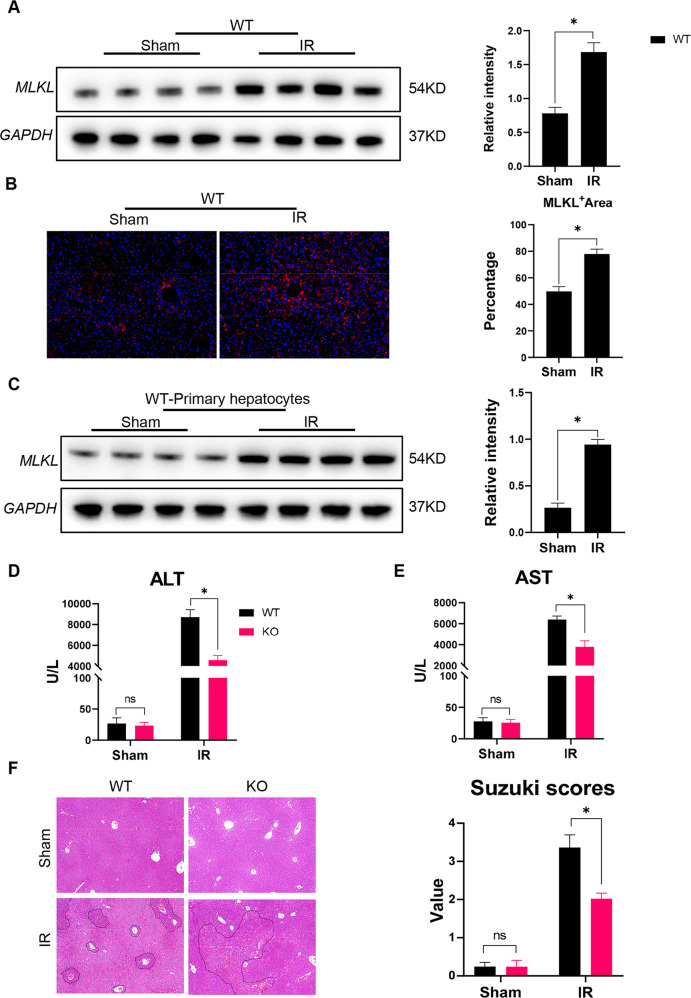
MLKL depletion protected livers against IR injury. WT mice were subjected to liver IR model or sham procedure. Liver tissues and primary hepatocytes were collected at 6 h post reperfusion. **A** Western blot analysis of MLKL and GAPDH in liver tissues. **B** Immunofluorescence staining of DAPI (blue) and MLKL (red) in liver tissues. **C** Western blot analysis of MLKL and GAPDH in primary hepatocytes. WT and MLKL KO mice were subjected to liver IR model or sham procedure. Liver tissues, blood samples were collected at 6 h post reperfusion. **D**, **E** Serum levels of ALT and AST. **F** H&E‐staining of liver sections; Suzuki scores based on H&E. *n* = 5 mice/group. All results represent at least two independent experiments. Values were expressed as mean ± SD. *p < 0.05.

We found that IR induced a significant increase in the expression of MLKL in the liver of WT mice (Fig. 1A, B). In order to further clarify the cells with increased MLKL expression after IR, primary hepatocytes were isolated and analyzed to further determine the changes of MLKL expression in hepatocytes. Results showed that MLKL expression was significantly increased in hepatocytes isolated from livers post IR (Fig. 1C). MLKL KO mice and WT controls were used to investigate the role of MLKL in regulating liver IR injury. Results showed that MLKL-deficient mice exhibited reduced liver IR injury, as evidenced by significantly lower levels of serum ALT and AST (Fig. 1D, E), better preserved liver architecture, and lower Suzuki’s scores (Fig. 1F). These findings suggest that MLKL deficiency ameliorates post-IR hepatocellular injury.

### MLKL depletion suppressed macrophage STING-mediated intrahepatic inflammation post-IR

**Fig. 2:**
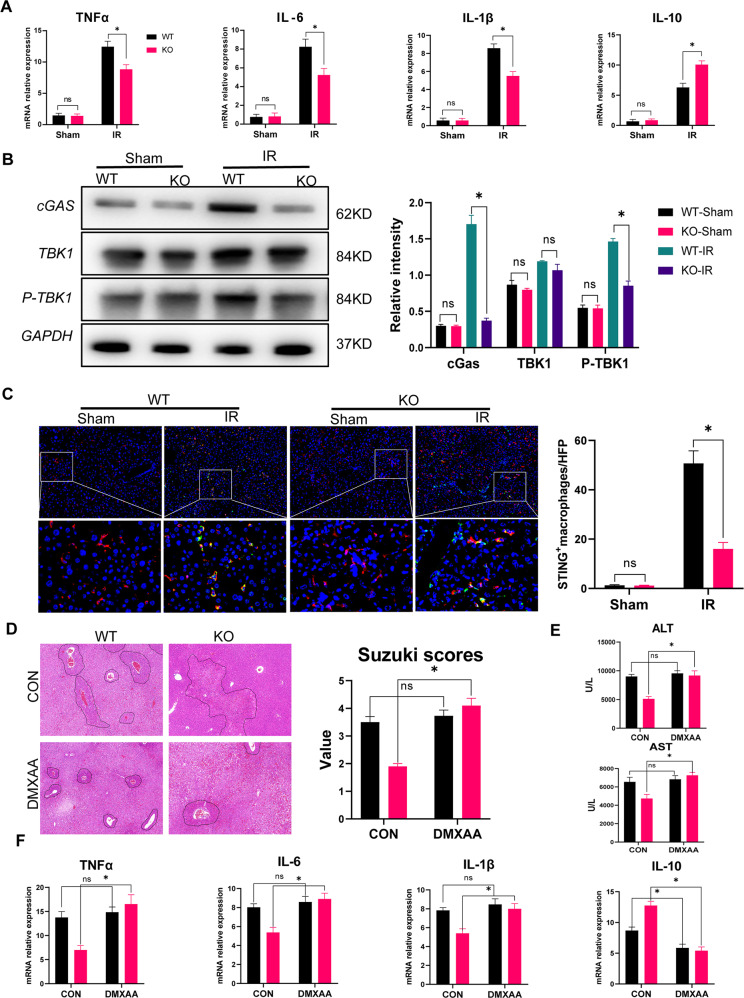
MLKL depletion attenuated inflammatory liver IR injury by suppressing macrophage STING activation. WT and MLKL KO mice were subjected to liver IR model or sham procedure. Liver tissues, blood samples and intrahepatic macrophages were collected at 6 h post reperfusion. **A** Gene expression of TNFα, IL-1β, IL-6 and IL-10 in liver tissues measured by qRT-PCR. **B** Expression of cGAS, TBK1, P-TBK1, and GAPDH in isolated intrahepatic macrophages measured by Western blot. **C** Immunofluorescence staining of DAPI (blue), F4/80 (red) and STING (green) in livers. WT and KO mice were pretreated with DMXAA (10 mg/kg) or PBS (Control) 3 h prior to ischemia. Mice were sacrificed 6 h poster reperfusion. **D** H&E‐staining of liver sections. **E** Serum levels of ALT and AST. **F** Gene expressions of TNFα, IL-1β, IL-6 and IL-10 in livers measured by qRT‐PCR. *n* = 5 mice/group. All results represent at least two independent experiments. Values were expressed as mean ± SD. *p < 0.05.

Intrahepatic inflammation was also compared between the WT and MLKL KO mice. Moreover, compared with that in the WT controls, the MLKL KO mice had significantly lower levels of the proinflammatory cytokines TNF-α, IL-6, and IL-1β but higher levels of the anti-inflammatory cytokine IL-10 (Fig. 2A). We further studied whether macrophage STING signaling is influenced by MLKL depletion and found that the macrophages isolated from the MLKL KO mice post-IR showed decreased cGAS and P-TBK1 protein levels (Fig. 2B). Moreover, the macrophages showed decreased STING activation, as indicated by F4/80 and STING double-staining (Fig. 2C).

Next, a STING-specific agonist, DMXAA, was used to determine the critical role of macrophage STING inhibition in protecting against liver IR injury by MLKL depletion. STING activation by DMXAA pretreatment abrogated the protective role of MLKL depletion, as evidenced by the worsened liver architecture (Fig. 2D), higher levels of serum ALT and AST (Fig. 2E), and increased intrahepatic inflammation (Fig. 2F). Together, these results indicate that MLKL depletion protects the liver against IR injury by suppressing the activation of macrophage cGAS-STING signaling.

### MLKL deficiency inhibited macrophage STING activation by reducing hepatocyte oxidative DNA damage

**Fig. 3:**
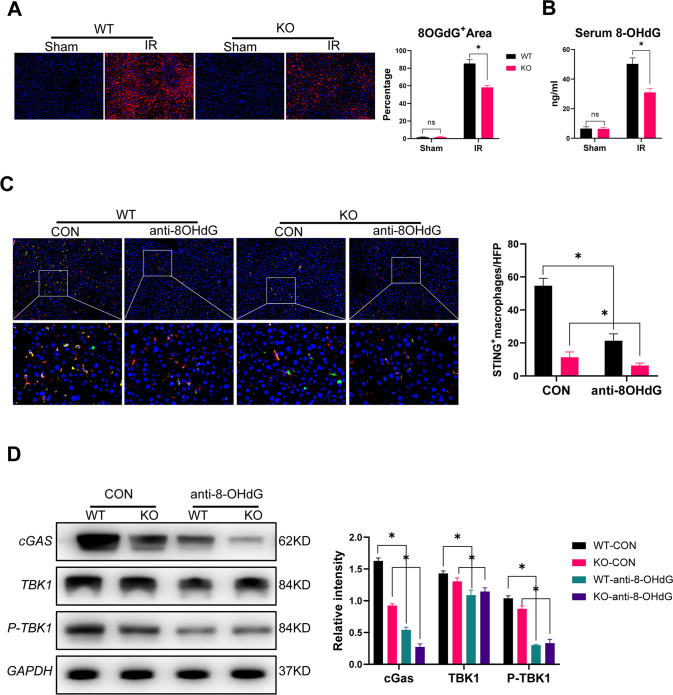
MLKL deficiency inhibited macrophage STING activation by reducing hepatocyte oxidative DNA damage. WT and MLKL KO mice were subjected to liver IR model or sham procedure. Liver tissues, blood samples and intrahepatic macrophages were collected at 6 h post reperfusion. **A** Immunofluorescence staining of DAPI (blue), 8-OHdG (red) in livers. **B** Serum levels of 8-OHdG. Mice were pretreated with anti-8-OHdG antibody (10 mg/kg) at 3 h prior to ischemia and sacrificed at 6 h post reperfusion. **C** Immunofluorescence staining of DAPI (blue), F4/80 (red) and STING (green) in livers. **D** Western blot analysis of cGAS, TBK1, P-TBK1, and GAPDH in isolated macrophages. *n* = 5 mice/group. All results represent at least two independent experiments. Values were expressed as mean ± SD. *p < 0.05.

Oxidative DNA injury contributes to the activation of cGAS-STING signaling [20]. Thus, we measured and compared intrahepatic oxidative DNA damage between the groups after IR. Moreover, the levels of 8-OHdG, a biomarker of oxidative DNA damage, significantly increased in the liver and serum post IR, which were decreased after MLKL KO (Fig. 3A, B). Moreover, inhibition of oxidative DNA damage by anti-8-OHdG treatment effectively suppressed macrophage STING activation in the WT and MLKL KO mice after IR (Fig. 3C, D).

**Fig. 4:**
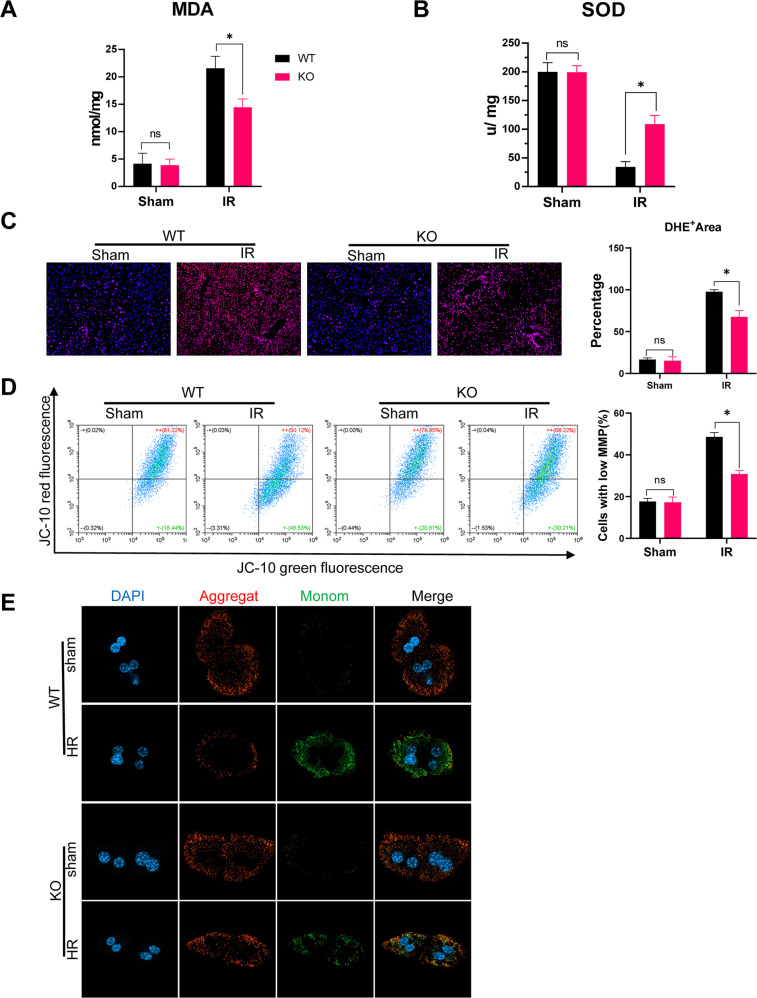
MLKL deficiency attenuated hepatocellular injury by suppressing oxidative stress. WT and MLKL KO mice were subjected to liver IR model or sham procedure. Levels of MDA (**A**) and SOD (**B**) in liver tissues. **C** Representative images of DHE (red) staining in liver tissues. **D** MMP detected by flow cytometry analysis of JC-10 in isolated primary hepatocytes. Primary hepatocytes were isolated from WT and KO mice and subjected to H/R model as described in the section of methods. **E** MMP measured by JC-10 staining. J-aggregates (Red), J-monomer (Green). *n* = 5 mice/group. All results represent at least two independent experiments. Values were expressed as mean ± SD. *p < 0.05.

The MLKL KO mice also showed decreased oxidative stress, as indicated by lower levels of MDA (Fig. 4A), higher levels of SOD (Fig. 4B), and lower DHE staining (Fig. 4C). Mitochondria are the main source of ROS and important target organs for oxidative injury. Moreover, MLKL depletion attenuated mitochondrial injury in hepatocytes post-IR, as indicated by the JC-10 analysis (Fig. 4D), which was further confirmed through in vitro analysis of H/R-stressed primary hepatocytes (Fig. 4E). Thus, MLKL deficiency suppresses hepatocyte oxidative DNA damage and subsequent macrophage STING activation during IR.

### MLKL depletion promoted PINK1-mediated mitophagy to reduce oxidative DNA damage in hepatocytes

**Fig. 5:**
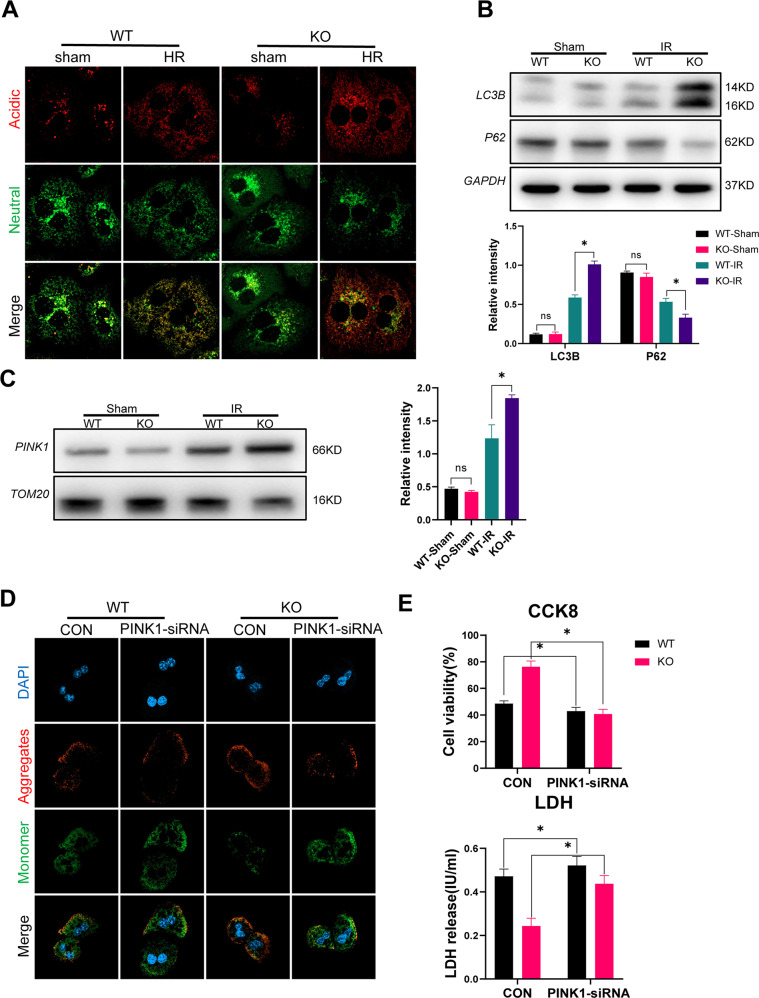
MLKL depletion promoted PINK1-mediated mitophagy to reduce oxidative DNA damage in hepatocytes. Primary hepatocytes were isolated from WT and MLKL KO mice and subjected to H/R model as described in the section of methods. **A** Representative confocal microscopy images of mt-mKeima-expressing primary hepatocytes, analyzed the pixel area in the red channel (acidic) and normalized to the signal in the green channel (neutral). **B** Western blot analysis of LC3B, P62 and GAPDH in primary hepatocytes. **C** Western blot analysis the mitochondrial protein of PINK1 and TOM20 in primary hepatocytes. Primary hepatocytes isolated from WT and KO mice were transfected with PINK1-siRNA or CON-siRNA (control) and subjected to H/R model. **D** Detection of MMP by JC-10 staining. J-aggregates (Red), J-monomer (Green). **E** Hepatocyte viability and injury/cell death were evaluated by CCK-8 assay and lactate dehydrogenase assay (*n* = 5). All results represent at least two independent experiments. Values were expressed as mean ± SD. *p < 0.05.

Mitophagy plays an important role in maintaining mitochondrial homeostasis and in regulating oxidative stress [21, 22]. Moreover, it has recently been reported that MLKL regulates autophagy [19]. Primary hepatocytes isolated from the WT and MLKL KO mice were subjected to the H/R model in vitro, and the mitophagic flux was investigated. An increased mitophagic flux was detected in the MLKL-deficient hepatocytes (Fig. 5A). The MLKL-deficient hepatocytes also demonstrated higher levels of LC3B and lower levels of P62 (Fig. 5B). Increased PINK1 levels were observed in the mitochondria (Fig. 5C). Blockade of PINK1 activation by PINK1-siRNA transfection aggravated mitochondrial injury (Fig. 5D), with increased cell injury/death and decreased cell survival (Fig. 5E) of H/R-stressed hepatocytes.

**Fig. 6:**
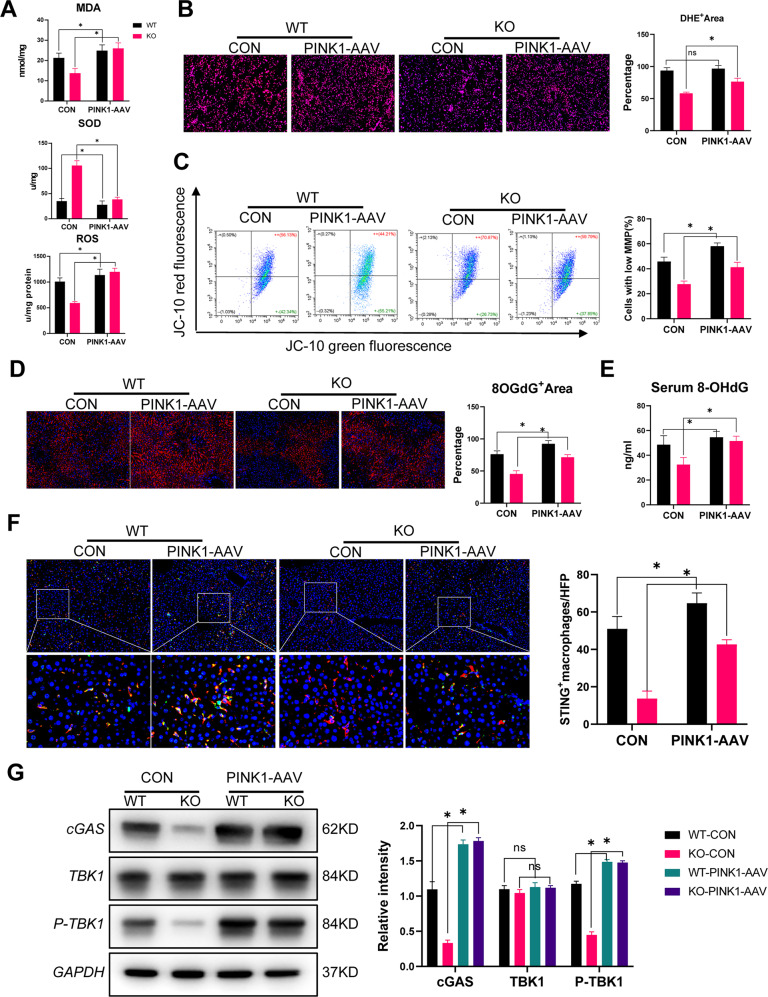
MLKL deficiency suppressed macrophage STING activation and inflammatory liver IR injury by activating mitophagy in hepatocytes. WT and MLKL KO were pretreated with AAV-PINK1-shRNA or AAV-CON-shRNA for 2 weeks and subjected to liver IR model. **A** Levels of MDA, SOD and ROS in liver tissues. **B** Representative images of DHE (red) staining in liver tissues. **C** MMP detected by flow cytometry analysis of JC-10 in isolated primary hepatocytes. **D** Immunofluorescence staining of DAPI (blue), 8-OHdG (red) in liver tissues. **E** Serum Levels of 8-OHdG. **F** Immunofluorescence staining of DAPI (blue), F4/80 (red) and STING (green) in liver tissues. **G** Western blot analysis of cGAS, TBK1, P-TBK1, and GAPDH in isolated intrahepatic macrophages. *n* = 5 mice/group. All results represent at least two independent experiments. Values were expressed as mean ± SD. *p < 0.05.

Finally, we examined the role of PINK1 inhibition in vivo. PINK1 inhibition significantly increased intrahepatic oxidative stress in the MLKL-KO mice (Fig. 6A, B). Hepatocytes from IR-stressed MLKL KO mice exhibited increased mitochondrial injury (Fig. 6C). Moreover, PINK1-AAV shRNA treatment effectively increased oxidative DNA damage in the MLKL KO mice, as evidenced by the increased levels of 8-OHdG in their liver tissues (Fig. 6D) and serum (Fig. 6E). This led to increased activation of cGAS-STING signaling in intrahepatic macrophages from the MLKL KO mice post-IR (Fig. 6F, G). In summary, these findings demonstrate that MLKL deficiency reduces hepatocyte oxidative DNA damage by promoting PINK1-mediated mitophagy, resulting in the suppression of macrophage cGAS-STING activation.

## Discussion

MLKL has been implicated in liver disease pathogenesis by regulating hepatocyte necroptosis [23]. However, the functions and molecular mechanisms of MLKL in hepatic IR injury require further investigation. In this study, we demonstrated an essential role of MLKL in modulating hepatocyte oxidative DNA injury and the subsequent macrophage proinflammatory response during ischemic liver injury.

We demonstrated that while IR injury induced MLKL upregulation in hepatocytes from mouse livers, MLKL depletion attenuated liver IR injury. Moreover, we observed decreased macrophage STING activation and intrahepatic inflammation in MLKL-deficient mice after IR. Mechanistically, reduced oxidative DNA damage due to increased mitophagy in hepatocytes contributes to the inhibition of macrophage cGAS-STING activation.

Liver IR is one of the most important causes of liver dysfunction and failure following partial hepatectomy or liver transplantation. Innate immune-mediated sterile inflammatory response plays an important role in the pathogenesis of liver IR injury [24]. Our previous studies as well as those of other researchers have highlighted the role of macrophages in regulating liver IR injury [25–27]. Moreover, DAMPs released from hepatocytes can be sensed by various pattern recognition receptors in macrophages [2]. Proinflammatory cytokine and chemokine secretion by activated macrophages triggers an inflammatory cascade and results in critical hepatocellular injury. Therefore, interventions targeting hepatocytes and macrophages may have potential therapeutic functions in liver IR injury.

Recent studies have shown that the cGAS-STING pathway plays an important role in various liver diseases [8, 9]. We previously showed that liver IR triggers macrophage STING activation [11], and STING inhibition attenuates different types of sterile inflammatory liver injuries, including liver IR [12, 28]. A recent study reported that STING induces liver IR injury by promoting calcium-dependent caspase 1-Gasdermin D processing in macrophages [29]. Suppression of STING signaling alleviates liver inflammation and IR injury [30]. It has also been reported that cGAS-mediated autophagy protects the liver against IR injury in a STING-independent manner [31]. In this study, we investigated the indirect role of MLKL signaling in regulating macrophage STING activation by regulating hepatocyte oxidative DNA injury.

Excessive oxidative stress can lead to cellular dysfunction and various liver diseases. Oxidized phospholipids promote ROS accumulation in hepatocytes to promote non-alcoholic steatohepatitis (NASH) development, which is ameliorated by the neutralization of oxidized phospholipids [32]. Ischemic insults cause accumulation of ROS and an imbalanced redox status in liver tissues, resulting in eventual liver damage [33]. Moreover, oxidative stress causes damage to DNA, which serves as an important DAMP that activates the proinflammatory immune response. Oxidative DNA damage in hepatocytes contributes to hepatocellular carcinoma formation in NASH [34]. ROS-mediated hepatocyte pyroptosis promotes the extracellular release of mtDNA and activation of macrophage STING during inflammatory liver injury [20]. We previously showed that oxidative stress induces mitochondrial injury and mtDNA release to promote cGAS-STING activation in macrophages during liver IR injury [12]. Drugs targeting increased repair of oxidative DNA damage may have therapeutic applications in various diseases [35, 36]. Nano-antioxidants effectively alleviate liver IR injury by scavenging ROS [37].

Mitophagy, an evolutionarily conserved cellular process that removes dysfunctional or superfluous mitochondria, is an important mechanism for maintaining mitochondrial homeostasis [21]. Insufficient autophagy or mitophagy contributes to the pathogenesis of multiple liver disorders including liver injury, NASH, fibrosis, and tumors [38]. DNA-dependent protein kinase catalytic subunit activates dynamin-related protein 1-related mitochondrial fission and inhibits FUN14 domain containing 1-required mitophagy to promote alcohol-related liver injury [39]. The interplay between mitophagy and ROS has been previously reported [22]. While mitophagy can be activated by ROS, it serves as a means of ROS clearance. Increased cytosolic mtDNA release owing to impaired mitophagy and increased oxidative mitochondrial injury may induce STING-mediated inflammatory injury. We previously showed that impaired mitophagy increases ROS-mediated hepatocyte pyroptosis to promote the extracellular release of mtDNA during acute liver injury [20]. We also found that defective mitophagy flux in aged macrophages aggravates liver IR injury by promoting cytosolic mtDNA release and STING-NLRP3 activation [12]. Failure to clear defective mitochondria via mitophagy drives autoimmune responses through mtDNA-dependent cGAS-STING activation [40]. Defective mitophagy promotes cytosolic mtDNA release to activate STING-NLRP3 signaling and increase sepsis severity [41].

Necroptosis is a form of regulated cell death mediated by RIP1, RIP3, and MLKL, which play important roles in development, inflammation, and diseases [42]. Necroptosis causes cell membrane rupture and the release of intracellular proinflammatory components [43, 44]. Deletion of RIPK3 or MLKL prevents the extracellular release of necroptotic DAMPs and inflammation [45]. RIPK3-MLKL-dependent necroptosis contributes to ischemia-induced cell death [14] and IR injury in steatotic livers [13, 15]. In contrast, a study also showed that RIPK3 and MLKL expression levels did not change in the liver IR model and Nec1s pretreatment did not protect the liver against IR injury [16]. We have previously shown that aging increases endoplasmic reticulum stress to aggravate hepatocyte necroptosis and liver IR injury [46]. A necroptosis-independent role of MLKL in the regulation of liver diseases has been revealed in recent studies. Emerging evidences have suggested the role of MLKL in regulating immune cells including macrophages. Increased MLKL expression in macrophages was found in ethanol-exposed livers, and myeloid MLKL deficiency exacerbated ethanol-induced steatosis and hepatocyte injury by impairing macrophage phagocytic capability [18]. Inhibition of RIPK1 improves NASH in an MLKL-dependent manner to increase mitochondrial respiration [47]. Critical roles of MLKL in regulating autophagy have been revealed recently. Activated MLKL inhibited autophagy flux in mouse dermal fibroblasts and HT-29 human colorectal cancer cells [48]. Overexpression of MLKL inhibited autophagy flux and activated inflammation in atherosclerosis, which was associated with a mechanistic target of rapamycin (mTOR)-dependent signaling pathway [49]. In contrast, MLKL activation by CAMK2 was found to promote autophagic flux in mouse Neuro-2a, L929, human HEK293 and HT29 cells in response to short-term starvation [50]. In livers, MLKL inhibits autophagy to promote hepatocyte cell injury and death during NASH [19]. Similarly, palmitic acid treatment induced the expression and translocation of MLKL to autophagosomes prior to the plasma membrane to inhibit autophagy, and MLKL deficiency restored autophagy activation in hepatocytes [51]. Therefore, dual functions of MLKL have been reported as an autophagy promoter or suppressor, depending on different types of cells, tissues and disease models. In this study, we found that MLKL activation suppressed hepatocyte mitophagy, resulting in macrophage STING activation.

In summary, our work identified, for the first time, the role of hepatocyte MLKL1 signaling in regulating macrophage STING activation during liver IR injury. These findings provide new mechanistic insights into the pathogenesis of liver IR injury and advance our knowledge of the necroptosis-independent functions of MLKL in the regulation of mitophagy and oxidative response. However, in the present study, the expression and role of MLKL in other infiltrating immune cells has not been investigated, which would be important and interesting for our further studies.

## Materials and methods

### Animals

Wild type (WT) male C57BL/6 J mice (8 weeks old) and MLKL KO male mice (8 weeks old) were housed at GemPharmatech Co, Ltd (China). MLKL KO mice were created by modifying the MLKL gene using CRISPR/Cas9 technology. Briefly, sgRNA was transcribed in vitro. Cas9 and sgRNA were microinjected into the fertilized eggs of C57BL/6 J mice. Fertilized egg Positive F0 mice were transplanted and confirmed by PCR and sequencing. Stable F1 generation mice were obtained by mating F0-positive mice with C57BL/6 J mice. All mice were maintained on a 12 h light/dark cycle with ad libitum access to standard water and chow and supplements under specific pathogen-free conditions. All animals received humane care according to a protocol approved by the Institutional Animal Care and Use Committee of Nanjing Medical University (number NMU08-092), and procedures for all animals complied with relevant legal and ethical requirements.

### Mouse hepatic IR model

Model of mouse partial hepatic warm IR injury was established as previously described [25]. Briefly, after successful anesthesia, an atraumatic clip was used to interrupt the arterial and portal venous blood supply to the cephalic lobes of the liver for 90 min and then the clip was removed to initiate hepatic reperfusion. Sham controls underwent the same procedure but without vascular occlusion. The mice were euthanized 6 h after reperfusion, and their blood samples and liver tissues were collected.

### Serum biochemical measurements and liver histopathology

Serum alanine aminotransferase (ALT) and aspartate aminotransferase (AST) levels were measured using an automatic chemical analyzer (Olympus, Japan). Portions of the liver specimens were fixed in 10% buffered formalin and embedded in paraffin. Sections of the paraffin-embedded liver tissues were stained with H&E. Severity of the hepatic IR was graded blindly from 0 to 4, according to the Suzuki criteria. No necrosis and no hyperemia/centrilobular globular changes were scored as 0, whereas severe hyperemia and >60% lobular necrosis were scored as 4.

### Isolation of liver cells and hypoxia/reoxygenation (H/R) model

As previously reported [52], livers were perfused in situ via the portal vein followed by 0.5% collagenase IV (Sigma, USA) and teased using 70 μm nylon mesh cell strainers (Biosharp, China). The liver cells were resuspended and then divided into non-parenchymal cells and hepatocytes. The non-parenchymal cells were replated for 30 min and the attached cells were considered to be macrophages.

Primary hepatocyte culture hypoxia/reoxygenation (H/R) patterns were cultured in a hypoxic incubator for 1 h [53]. The culture conditions were then adjusted to normal for 6 h. The cells and supernatants were used in the subsequent experiments.

### Detection of cell survival and death

Hepatocyte viability was quantified using the Cell Counting Kit-8 assay (Yeasen Biotechnology, China). Moreover, hepatocyte cytotoxicity was determined by measuring lactate dehydrogenase activity (Promega, USA) according to the manufacturer’s protocol.

### PTEN‐induced kinase1(PINK1) knockdown

For knockdown of PINK1 in vivo, adeno-associated virus (AAV) vectors carrying PINK1 short hairpin RNA (shRNA) or control shRNA (GeneChem, China) were administered intravenously at a dose of 4 × 10^11^ PFU per mouse into the tail vein of 8-week-old male WT or MLKL KO mice. After two weeks, all the mice were established as liver ischemia-reperfusion models.

In vitro, primary hepatocytes were prepared and transiently transfected with PINK1 small interfering RNA (siRNA) or control siRNA (GeneChem, China) using Lipofectamine 3000 (Thermo Fisher Scientific, USA). The PINK1 siRNAs targeted the following sequences: sense, 5′GCUGCAAUGCCGCUGUGUA3′, antisense, 5′ UACACGCGGCAUUGCAGC3′. For si-NC, the sequences were as follows: sense, 5′GAUCAUACGUGCGAUCAGA3′, antisense, 5′UCUGAUCGCACGUAUGAUC3′.

### STING and 8-hydroxy-2deoxyguanosine (8-OHdG) signaling intervention

DMXAA (MedChemExpress, USA), a STING-specific agonist, was injected intraperitoneally at a dose of 10 mg/kg per mouse, 3 h before the onset of liver ischemia.

To study the effects of 8-OHdG inhibition on hepatic IR, 8-week-old WT or MLKL KO male mice were intraperitoneally injected with mouse monoclonal anti-8-OHdG antibody (GeneTex, USA) at a dose of 10 mg/kg per mouse, 3 h before the onset of liver ischemia.

### Immunohistochemical staining

For tissue immunofluorescence analysis, the liver tissue sections of the mice (4 μm thick) were stained with the indicated primary antibodies MLKL (Proteintech, China), mouse EGF-like module-containing mucin-like hormone receptor-like1 (F4/80) (Cell Signaling Technology, USA), STING (Cell Signaling Technology, USA), and 8-OHdG (Santa Cruz Biotechnology, USA) and incubated overnight at 4 °C. The sections were then washed twice and incubated with the secondary Cy3-conjugated goat IgG (Sigma, USA) and FITC-conjugated IgG (Sigma, USA) according to the manufacturer’s instructions for 30 min at 37 °C. In addition, 4′,6‐diamidino‐2‐phenylindole (Beyotime Biotechnology, China) was used for nuclear staining. Representative images were captured and viewed using a confocal microscope (Zeiss).

### Western blotting

Proteins of the liver tissue or cells were extracted prepared. Antibodies against GAPDH, MLKL, cGAS, TANK-binding kinase1 (TBK1), phosphor-TANK-binding kinase1 (P-TBK1) (Cell Signaling Technology, USA), PINK1, P62, LC3B (Proteintech, China), and TOM20 (Santa Cruz Biotechnology, USA) were used. Furthermore, after 2 h of incubation of the membranes with the appropriate horseradish peroxidase (HRP)-conjugated secondary antibody, the bands were detected using Immobilon ECL Ultra Western HRP substrate (Vazyme Biotechnology, China) and images were taken using the Vilber chemiluminescence imaging system (Vilber Bio Imaging, France, FUSION-FX6.EDGE V.070). Density determination was performed using ImageJ software to determine changes in protein expression.

### Quantitative real-time-PCR

Total RNA was extracted from the liver tissues and cells using an RNA extraction kit (Invitrogen, USA). Reverse transcription of the RNA into complementary DNA was performed using an RR047A PrimeScript RT Reagent Kit with gDNA Eraser (TaKaRa, Japan). Quantitative real-time PCR was performed and repeated thrice. The expression of target genes was normalized to that of GAPDH. The primer sequences used for the qPCR are listed in Supplementary Table 1.

### Dihydroethidium (DHE) staining

Fluorescent probes of DHE were used to detect intracellular reactive oxygen species (ROS) in the liver tissues. Cryosections of the liver tissues were incubated with 10 μM DHE for 30 min at 37 °C. Fluorescence was detected at 488 nm excitation and 525 nm emission wavelengths using a confocal microscope.

### Measurement of malondialdehyde (MDA), superoxide dismutase (SOD), and ROS levels

The levels of MDA, SOD, and ROS in the liver tissues were estimated using commercial kits. The liver tissues were washed with PBS, homogenized in lysis buffer, and sonicated. After sonication, the lysed tissues were centrifuged (10,000 × *g*, 10 min) to remove debris and to retain the supernatant. The contents of MDA, SOD, and ROS in the supernatant were determined using a microplate reader. In addition, MDA, SOD, and ROS levels were normalized to protein concentration.

### Measurement of mitochondrial membrane potential (MMP)

MMP was detected by flow cytometry and immunofluorescence staining using a JC-10 assay kit (Yeasen Biotechnology, China). As a lipophilic dye, it is concentrated in healthy mitochondria to form reversible red fluorescent aggregates and is released into the cytoplasm to form green fluorescent monomers when mitochondria are damaged, resulting in a decrease or disappearance of membrane potential. A reduction in the red signal indicates a decrease in the MMP.

### Detection of mitophagy

Primary hepatocytes from the mouse liver tissues were transduced with adenovirus-coated-COX8-mt-mkeima (ObiO Technology Corp., Ltd, China) for 24 h to assess the mitophagy levels. Moreover, mt-keima can bind to the mitochondrial matrix, and its fluorescence varies in different pH environments. An increase in red signal indicates a higher level of mitophagy flux. The levels of mitophagy were measured by the pixel area in the red channel (acidic) and normalized to the signal in the green channel (neutral).

### Statistical and analysis

Results are shown as the mean ± SD and represent at least two independent experiments. Statistical analysis was performed using Student’s test between two groups and one-way analysis of variance between multiple groups followed by Bonferroni’s post hoc test. All analyses were performed with Graphpad8.0. *p*‐value < 0.05 (two‐tailed) was considered statistically significant.

## Supplementary information

Table S1

Original Data File

## Data Availability

The data of this study are available from the corresponding author on reasonable request.
