# Supplementary material for: MLKL deficiency attenuated hepatocyte oxidative DNA damage by activating mitophagy to suppress macrophage cGAS-STING signaling during liver ischemia and reperfusion injury
Source: Cell Death Discov. 2023 Feb 10;9:58. doi: 10.1038/s41420-023-01357-6 (PMC9918524; doi:10.1038/s41420-023-01357-6)
Supplement: Supplementary file 1 — Table S1 [file 41420_2023_1357_MOESM1_ESM.docx]

**Supplementary table 1**

List of primer used for RT-PCR.

| **Gene Forward Primer Reverse Primer** |
| --- |
| GAPDH AGGTCGGTGTGAACGGATTTG GGGGTCGTTGATGGCAACA |
| TNFα CAGGCGGTGCCTATGTCTC CGATCACCCCGAAGTTCAGTAG |
| IL-6 CTGCAAGAGACTTCCATCCAG AGTGGTATAGACAGGTCTGTTGG |
| IL-10 GCTGGACAACATACTGCTAACC ATTTCCGATAAGGCTTGGCAA |
| IL-1β GAAATGCCACCTTTTGACAGTG TGGATGCTCTCATCAGGACAG |
