# Supplementary material for: MLKL deficiency attenuated hepatocyte oxidative DNA damage by activating mitophagy to suppress macrophage cGAS-STING signaling during liver ischemia and reperfusion injury
Source: Cell Death Discov. 2023 Feb 10;9:58. doi: 10.1038/s41420-023-01357-6 (PMC9918524; doi:10.1038/s41420-023-01357-6)
Supplement: Supplementary file 2 — Original Data File [file 41420_2023_1357_MOESM2_ESM.docx]

**

Fig1A: MLKL**

**

 Fig1A: GAPDH**

**Fig1C: MLKL Fig1C: MLKL**







**Fig2B: TBK1 Fig2B: GAPDH**







**Fig2B: cGAS Fig2B: PTBK1**







**Fig3D: GAPDH**





**Fig3D: cGAS Fig3D: P-TBK1**








**Fig3D: TBK1 Fig5B: LC3B**







**Fig5B: GAPDH Fig5B: P62**







**Fig5C: TOM20 Fig5C: PINK1**







**Fig6G: TBK1 Fig6G: GAPDH**







**Fig6G: cGAS**

**Fig6G: PTBK1**
